# Modulation of Intrinsic Brain Connectivity by Implicit Electroencephalographic Neurofeedback

**DOI:** 10.3389/fnhum.2020.00192

**Published:** 2020-06-23

**Authors:** Olga R. Dobrushina, Roza M. Vlasova, Alena D. Rumshiskaya, Liudmila D. Litvinova, Elena A. Mershina, Valentin E. Sinitsyn, Ekaterina V. Pechenkova

**Affiliations:** ^1^Third Neurological Department, Research Center of Neurology, Moscow, Russia; ^2^International Institute of Psychosomatic Health, Moscow, Russia; ^3^Department of Psychiatry, University of North Carolina, Chapel Hill, NC, United States; ^4^Davydovsky Public Clinical Hospital, Moscow, Russia; ^5^Radiology Department, Federal Center of Treatment and Rehabilitation, Moscow, Russia; ^6^Medical Research and Educational Center, Lomonosov Moscow State University, Moscow, Russia; ^7^Laboratory for Cognitive Research, National Research University Higher School of Economics, Moscow, Russia

**Keywords:** neurofeedback, functional magnetic resonance imaging, resting-state fMRI, intrinsic brain connectivity, salience network

## Abstract

Despite the increasing popularity of neurofeedback, its mechanisms of action are still poorly understood. This study aims to describe the processes underlying implicit electroencephalographic neurofeedback. Fifty-two healthy volunteers were randomly assigned to a single session of infra-low frequency neurofeedback or sham neurofeedback, with electrodes over the right middle temporal gyrus and the right inferior parietal lobule. They observed a moving rocket, the speed of which was modulated by the waveform derived from a band-limited infra-low frequency filter. Immediately before and after the session, the participants underwent a resting-state fMRI. Network-based statistical analysis was applied, comparing post- vs. pre-session and real vs. sham neurofeedback conditions. As a result, two phenomena were observed. First, we described a brain circuit related to the implicit neurofeedback process itself, consisting of the lateral occipital cortex, right dorsolateral prefrontal cortex, left orbitofrontal cortex, right ventral striatum, and bilateral dorsal striatum. Second, we found increased connectivity between key regions of the salience, language, and visual networks, which is indicative of integration in sensory processing. Thus, it appears that a single session of implicit infra-low frequency electroencephalographic neurofeedback leads to significant changes in intrinsic brain connectivity.

## Introduction

An important clinical application of modern neuroscience research is the development of neuromodulation. In particular, neurofeedback is increasingly used to treat developmental disorders, headaches, anxiety and depression, schizophrenia, and other diseases (Marzbani et al., 2016). During neurofeedback, a computerized training is performed based on a neurophysiological signal, such as electroencephalography (EEG) or functional magnetic resonance imaging (fMRI). It is supposed that after several sessions an improved brain functional state would lead to enhanced everyday life performance. Potential advantages of neurofeedback in comparison with pharmacotherapy include more durable effects and a lack of drug-related complications (Van Doren et al., 2019).

Currently, there are ongoing debates around neurofeedback. Some researchers wonder whether the observed improvements are due to the neurophysiological intervention *per se* or are related to nonspecific effects, such as behavioral or suggestion therapy, and call for proper controlled studies (Thibault et al., 2018; Lubianiker et al., 2019; Sorger et al., 2019). Other authors point to several methodological barriers interfering with an evidence-based evaluation of neurofeedback (Ioannides, 2018; Pigott et al., 2018; Sorger et al., 2019). Meanwhile, a recent high quality randomized controlled study demonstrated the efficacy of slow cortical potential EEG neurofeedback in children with attention-deficit and hyperactivity disorder (Strehl et al., 2017). In addition to evaluating its efficacy, it is important to continue the research targeted at describing the mechanisms of neurofeedback and developing relevant neuroscientific models.

In the current study, we investigated the neural basis of implicit infra-low frequency EEG neurofeedback—a modality with poorly understood mechanisms that is extensively used in practice (Legarda et al., 2011; Alvarez et al., 2013; Othmer et al., 2013). During an implicit neurofeedback session, the clients do not pursue any goal to change (increase or decrease) the feedback signal; no intentional activity is required. Feedback information is deemed to be utilized by self-regulatory neural networks to optimize brain function (Othmer, 2011). The mechanisms of implicit neurofeedback may be explained in light of the natural role of feedback in neurodevelopment (Ioannides, 2018) and also in light of the predictive models of nervous activity since feedback is supposed to serve for augmentation of internal neural models (Wolpert et al., 1995; Kleckner et al., 2017). Despite the passive nature of the “training” paradigm, pronounced neurophysiological and clinical effects of implicit neurofeedback sessions have been described (Alvarez et al., 2013; Ramot et al., 2016; Zhigalov et al., 2016; Grin-Yatsenko et al., 2018).

**Figure 1:**
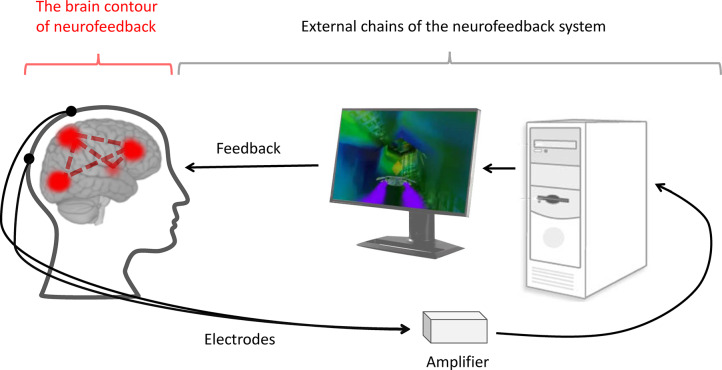
The neurofeedback system. During neurofeedback, an artificial regulatory system is formed. It includes external chains, such as the electrodes, amplifier, computer, software and feedback representation (black lines), and internal chains—the brain contour of the neurofeedback (red lines). The latter consists of the brain networks responsible for processing, controlling, and utilizing the feedback information.

**Figure 2:**
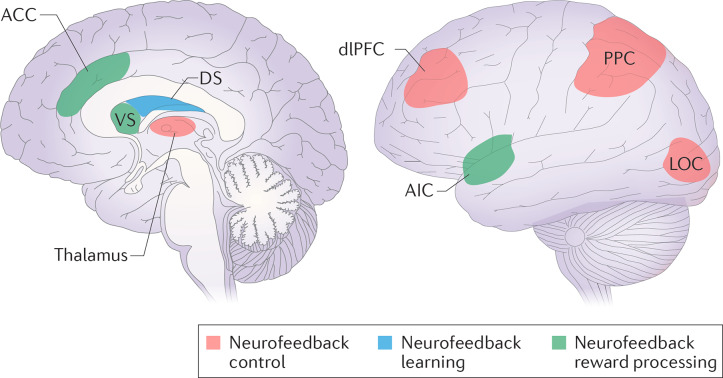
Neurofeedback control, learning and reward processing networks. The regions from the frontoparietal control network (dorsolateral prefrontal cortex—dlPFC; posterior parietal cortex—PPC), in cooperation with the task-relevant modality sensory cortex (lateral occipital cortex—LOC), are supposed to be responsible for neurofeedback control. Task-related learning involves the dorsal striatum (DS). The reward may be processed either consciously by the salience network (the anterior cingulate cortex—ACC, the anterior insular cortex—AIC), or unconsciously by the ventral striatum (VS). Reprinted by permission from Springer, from Sitaram et al. (2017).

In designing the study, we relied on existing data on the modulation of brain connectivity by neurofeedback in other modalities, including multiple studies of explicit fMRI neurofeedback (Amiez et al., 2012; Emmert et al., 2016; Sitaram et al., 2017; Paret et al., 2018, 2019), a single study of implicit fMRI neurofeedback (Ramot et al., 2016) and a few studies of explicit EEG neurofeedback (Ros et al., 2013; Kluetsch et al., 2014). To organize the data, we decided to separate two phenomena. First, the influence of explicit neurofeedback on brain connectivity may be related to the formation of neuronal assemblies accomplishing the task, which we would subsequently term the “neurofeedback neural contour” (see Figure 1). We suppose that implicit neurofeedback may engage a similar neural contour even in the absence of the task. When evaluating the neurofeedback contour, we mainly relied on the review by Sitaram et al. (2017): the control over neurofeedback is believed to be accomplished by the Frontoparietal Control Network (FPCN), in cooperation with the areas of the sensory cortex relevant to the sensory modality of the feedback, and the thalamus; meanwhile the salience network and striatum take part in the perception of reward and error monitoring (see Figure 2). This view is supported by an fMRI neurofeedback metanalysis by Emmert et al. (2016), and, importantly, by the only study utilizing implicit neurofeedback protocol (Ramot et al., 2016). We also relied on the data of the more recent studies by Paret et al. (2018, 2019), who described the role of the ventromedial prefrontal cortex (vmPFC) and the orbitofrontal cortex (OFC) in the neurofeedback contour. Regarding the second phenomenon, modifications in brain networks after neurofeedback may underly its desired effects, such as relaxation and concentration. For example, alpha desynchronization EEG training has been shown to result in increased connectivity in the salience and default-mode networks (Ros et al., 2013; Kluetsch et al., 2014).

Following these two *a priori* defined directions, we evaluated the neurofeedback neural contour and searched for alterations in brain networks that may mediate the behavioral effects. The assessment was performed using resting-state functional MRI (rsfMRI). An important advantage of this approach is that rsfMRI is currently a gold standard method to reveal intrinsic connectivity networks, while EEG neurofeedback is a widely available option that can be tolerated by almost any patient, including children with severe developmental delays, and its possible applications are especially wide in the case of an implicit paradigm. Investigation of this relatively simple technique using rsfMRI would allow the assessment of its lasting effects on brain circuits with great spatial precision and further translation of the growing data on functional brain connectivity to clinical applications.

## Materials and Methods

### Participants and Experimental Design

A total of 53 volunteers (22 males) entered the study. The following inclusion criteria were used: age range from 18 to 44 years, right-handed, no history of neurological or psychiatric disorders, and no general contraindications for MRI. The participants were mainly recruited among students and graduates of the Moscow State University and Higher School of Economics; they received no payment. The sample consisted of Caucasian volunteers aged 27 ± 6 years. The participants were randomized into one of two groups: neurofeedback (NF, *n* = 27) or sham-neurofeedback (sham-NF, *n* = 26). The data of one participant from the NF group were excluded from the analysis due to excessive head motion, resulting in 26 participants in each group. The study was approved by the Inter-University Ethics Board of Moscow. Before the experiment, written informed consent was obtained from each participant.

**Figure 3:**
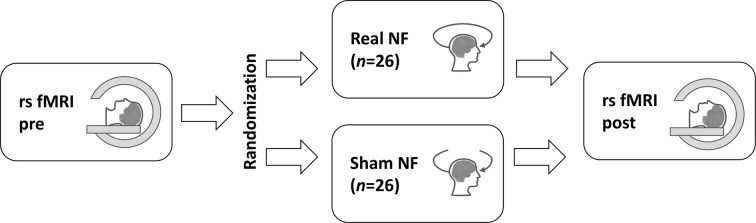
The study design. The resting-state fMRI (rs fMRI) was performed twice: before (pre) and after (post) the real or sham neurofeedback (NF) session. The study participants were randomly assigned to the groups.

The participants and all the experimenters except for the one providing the neurofeedback were blinded to the group randomization order. The study consisted of three subsequent phases: initial resting-state fMRI scan; NF or sham-NF EEG session (outside the scanner); and second fMRI scan (see Figure 3). The NF (or sham-NF) session was performed outside of the scanner due to the incompatibility of the EEG neurofeedback equipment with MRI. This outside of scanner design also has some benefits: it is less demanding for participants, so the risk of falling asleep during the scanning is reduced; and it is more similar to clinical practice. The intervals between any two subsequent phases did not exceed 20 min. The participants were asked not to engage in any side activity during the entire experiment.

For a *post hoc* analysis of the effects of repeated scanning, we used an open NYU CSC TestRetest resource, downloaded from https://www.nitrc.org/projects/nyu_trt (Zuo et al., 2010). This dataset consisted of resting-state EPI-images of 25 participants (10 males, from 22 to 49 years old), acquired during rest with a time delay of about 30 (<45) minutes between the two sessions.

### Neurofeedback Procedure

The participants underwent a 30-min session of either real or sham infra-low frequency neurofeedback provided by the same experimenter. EEG signals were recorded from Ag/AgCl sintered electrodes positioned at the P4 and T4 sites according to a 10-20 system, while the ground electrode was placed on the forehead. This site is the usual starting point of infra-low frequency neurofeedback protocols with minimal risk of side effects (Othmer, 2017). The skin was prepared with NuPrep abrasive paste, and the electrodes were fixed with 10-20 conductive paste to reach impedance below 5 kOhm. The EEG signal was recorded with a NeuroAmp (Corscience GmbH) DC-amplifier, sampled with 1 K samples per second, filtered and down-sampled to 250 samples per second and 32-bit resolution for further processing in the Cygnet biofeedback software (BEE Medic GmbH).

During the session, participants watched the InnerTube neurofeedback game (Somatic Vision Inc.) featuring a rocket moving through tunnels. The speed of the rocket was governed in real-time by the infra-low frequency band-limited waveform of the EEG signal (see Figure 1). The infra-low frequency domain (*f* < 0.1 Hz) is thought to reflect the slow dynamics of cortical activation, is a real-time representation of slow cortical potentials (Grin-Yatsenko et al., 2018). Infra-slow fluctuations have been shown to correlate with resting-state network dynamics in fMRI (Hiltunen et al., 2014; Haufe et al., 2018). In the sham group, a simulated signal was used. The simulated signal was created by a random number generator, and the spectral power density was shaped to match that of a typical EEG.

All participants received the same written explanation of the experimental procedure. They were instructed to sit in front of the monitor and watch the visualization. It was explained that in case they are randomized into the NF group, the speed of the rocket is influenced by the brain signal, but otherwise, it is not. There was no instruction to voluntarily control the rocket. The higher (or lower) speed of the rocket was not interpreted to be beneficial. At the end of the entire experimental session (i.e., after the second MRI scan), the randomization order was disclosed to the participants. Before the disclosure, they were asked if they had any idea about which group they belonged to, and none reported they had.

### fMRI Acquisition and Preprocessing

MRI was performed with the 3.0T GE Discovery MR750 scanner located at the Federal Center of Treatment and Rehabilitation (Moscow) using a standard 16-channel head, neck, and spine array coil. Each MRI session consisted of an anatomical scan and a 10-min resting-state functional session, where the participants were asked to close their eyes, restrict themselves from systematic voluntary intellectual activity such as counting or solving problems, and to lie as still as possible. Structural T1-weighted images were acquired with a 3D FSPGR sequence (180 sagittal 1-mm slices). Three-hundred functional T2*-weighted images were acquired with a gradient-echo echo-planar imaging sequence with the following parameters: voxel size = 3 × 3 × 3 mm^3^; matrix size = 64 × 64 × 42; TR = 2,000 ms; TE = 30 ms; FA = 77°; FoV = 192 × 192 mm. The field of view covered the whole brain, and the slices were oriented parallel to the AC/PC plane. Four extra functional volumes were acquired at the start of the session and discarded by the scanner software to prevent the usage of artifactual data acquired before the magnetic equilibrium was reached.

Image preprocessing was performed with Statistical Parametric Mapping (SPM8) package^1^ (RRID:SCR_007037) and included slice-timing correction, realignment, co-registration of the average structural image from the pre- and post- sessions to the average of functional images, segmentation of the average structural image into tissue images (gray matter, white matter, and cerebrospinal fluid volumes), spatial normalization into standard Montreal Neurological Institute (MNI) space and spatial smoothing using a Gaussian kernel of 8 mm full width at half maximum. Two structural volumes were coregistered and averaged before the coregistration of the structural to functional images and segmentation, and the functional images from the two sessions for each participant were realigned together without session concatenation.

Since the rsfMRI data are extremely sensitive to participant head motion and physiological (respiratory, cardiac) artifacts, and even subtle motion may result in spurious artifactual correlations between voxel time series (Power et al., 2012), several extra preprocessing steps were performed with the help of the CONN toolbox, Version 17a^2^ (RRID:SCR_009550). First, artifacts were addressed by the motion-scrubbing procedure (ART toolbox^3^, RRID:SCR_005994) which involved the detection of outlier scans characterized by head displacement greater than 0.9 mm in any direction or deviation of the image global intensity by more than five standard deviations from the session mean. To preserve the temporal structure of the data, the outliers were further deweighted at the modeling stage, and the number of outliers was included in the model as a subject-level covariate to account for possible individual differences in head motion. Second, we applied the anatomical component-based analysis aCompCor (Behzadi et al., 2007), a method that involves regressing out the principal components of the BOLD-signal estimated from the white matter and the cerebrospinal fluid volumes in each participant. This signal is considered to be an empirical measure of noise. Third, six residual head motion parameters and their derivatives were regressed out from the signal. Finally, temporal lowpass and highpass frequency filters were applied to the data restricting the analysis to frequencies of 0.008–0.12 Hz. We selected a frequency band wider than the default range of 0.008–0.09 Hz following the cutoff value used by Scheinost et al. (2013) to include all the infra-slow fluctuations, which have been shown to have common dynamics in the EEG and fMRI signals (Hiltunen et al., 2014).

The NYU CSC TestRetest dataset was acquired by the researchers from New York University with the use of a Siemens Allegra 3.0 Tesla scanner (Zuo et al., 2010). We analyzed the two subsequent resting state (eyes open) scans, which were done 30–45 min apart. Each of them consisted of 197 contiguous EPI functional volumes (TR = 2,000 ms; TE = 25 ms; FA = 90°, 39 slices, matrix = 64 × 64; FOV = 192 mm; acquisition voxel size = 3 × 3 × 3 mm), and a high-resolution T1-weighted magnetization prepared gradient echo sequence (MPRAGE, TR = 2,500 ms; TE = 4.35 ms; TI = 900 ms; FA = 8°; 176 slices, FOV = 256 mm). The pre-processing and post-processing of the NYU CSC TestRetest dataset followed the same protocol used with the original data.

### Functional Connectivity Analysis

#### *A priori* Assumptions and General Analytic Strategy

**Figure 4:**
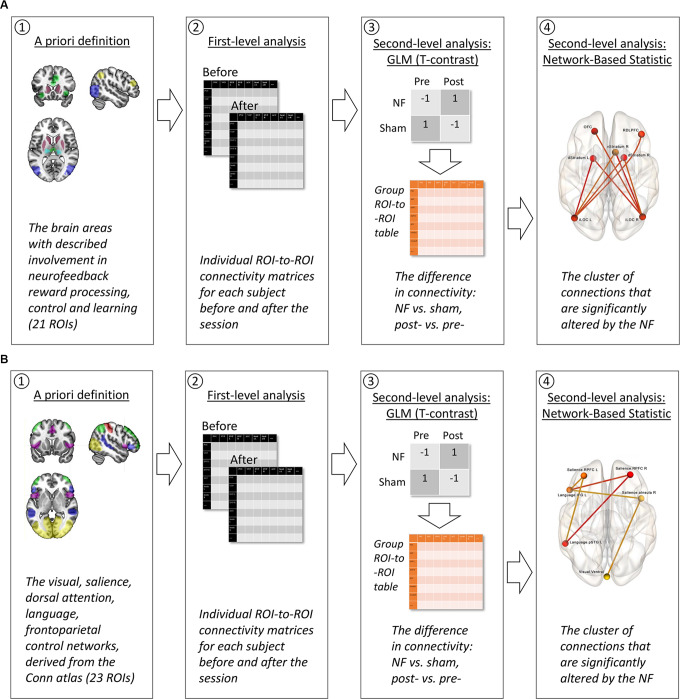
The strategy of fMRI data analysis. **(A)** Neurofeedback contour hypothesis. (1) *A priori*, we selected the ROIs representing brain areas suspected to be involved in neurofeedback reward processing, control, and learning, following the available studies of fMRI neurofeedback and explicit electroencephalography (EEG) neurofeedback which were reviewed by Sitaram et al. (2017). (2) During the first-level analysis, the fMRI time series from these 21 ROIs were used to conduct individual subject ROI-to-ROI connectivity matrices for each of the two scans: before and after the neurofeedback (NF) or sham-NF session. (3) The second-level analysis included two steps. A general linear model (GLM) with T-contrast “NF vs. sham, post- vs. pre-” allowed us to discriminate the changes in ROI-to-ROI connectivity resulting from the real but not the sham NF. At this step, a group ROI-to-ROI matrix of connectivity differences assessed with the contrasts was calculated. (4) Next and finally, this group ROI-to-ROI matrix entered the network-based statistic (NBS) analysis. During the NBS, a multiple comparisons correction was applied, and a cluster of significant connections between the ROIs was revealed. **(B)** Attention hypothesis. (1) *A priori*, we selected all the ROIs for the attentional networks (Visual, Salience, Dorsal Attention, Language, Frontroparietal Control Network) from the CONN networks atlas, following the described clinical effects of the infra-low frequency neurofeedback from the T4P4 site: increased awareness to the internal and external stimuli, and sensory integration (Othmer, 2017). Steps 2–4 were identical to the neurofeedback contour hypothesis (**A**).

Relying on the existent literature (see “Introduction” section), we classified the possible influences of neurofeedback on intrinsic brain connectivity into two phenomena, which resulted in two lines of analysis: the neurofeedback contour hypothesis (see Figure 4A) and the attention hypothesis (see Figure 4B).

In the first line of analysis (see Figure 4A) we aimed to find the brain contour underlying the implicit neurofeedback process, using as a starting point the results of existing research on explicit EEG/fMRI and implicit fMRI neurofeedback (Amiez et al., 2012; Emmert et al., 2016; Sitaram et al., 2017; Paret et al., 2018, 2019; see Figure 2). We assumed that the transient neuronal assemblies related to the neurofeedback might still be detectable immediately after the training. Areas with a suspected role in neurofeedback control, learning, and reward processing were used as regions of interest (ROI) for the ROI-to-ROI analysis. The BOLD-signal time series from these ROIs were extracted and used to compute the ROI-to-ROI correlation matrices for each subject (first level). Next, at the group level, a general linear model (GLM) was applied to discriminate the effects related to the real but not sham neurofeedback. Finally, a network-based statistic (NBS) approach was used to correct for multiple comparisons and to identify the clusters of interrelated connections between ROIs participating in the neurofeedback contour (Zalesky et al., 2010). NBS analysis, based on graph theory, is targeted at the identification of integrated networks rather than particular connections, which is relevant to the nature of neurofeedback.

In the second line of analysis (see Figure 4B) we aimed to identify changes in the intrinsic connectivity underlying the specific desired effects of the applied neurofeedback modality, i.e., an increase in awareness. In the current study, we utilized an infra-low frequency protocol from the T4P4 site—bipolar montage with electrodes over the right middle temporal gyrus and the right inferior parietal lobule (Koessler et al., 2009). Such training is known to induce a calm and alert mental state with an increased awareness for both external (environment) and internal (body) stimuli, and to promote sensory integration (Othmer, 2017). Following the described clinical effects which may be qualified as attention-related effects (selection of stimuli, allocation of mental resources, and information integration are the classical examples of attention processes; e.g., Styles, 1997), we performed an exploratory search for modifications in task-positive attention-related intrinsic connectivity networks. The analysis followed the same procedure as in the evaluation of the neurofeedback contour hypothesis.

Finally, we performed an exploratory search for local alterations in connectivity with a hypothesis-free intrinsic connectivity contrast (ICC) analysis (Martuzzi et al., 2011)—see “Intrinsic Connectivity Contrast Analysis” section.

Functional connectivity analysis was performed with the CONN toolbox.

#### ROI Analysis: Definition of the ROI

When selecting ROIs for the neurofeedback contour hypothesis (see Table 1), we considered the review by Sitaram et al. (2017) as well as the data from more recent studies (Paret et al., 2018, 2019). The latter two articles reported exact coordinates for the areas in the vmPFC and the left OFC, which we relied on (Paret et al., 2018, 2019). Since Sitaram et al. (2017) provide accumulated evidence on the neurofeedback contour components rather than exact coordinates (see Figure 2), we outlined the corresponding ROIs based on the Harvard Oxford Atlas (Desikan et al., 2006), a study by Seeley et al. (2007) differentiating salience and executive networks (Seeley et al., 2007) and parcellation of the thalamus by Najdenovska et al. (2018). For all cortical regions specified by peak coordinates, the ROIs were constructed as 10-mm radii spheres around the given coordinates. The ROIs from the Harvard-Oxford Atlas and thalamus parcellation followed the exact outlines specified in the atlases.

**Table 1:** Regions of interest (ROIs) within the proposed neurofeedback contour (Hypothesis 1).

ROI		MNI coordinates (x, y, z) of the center of mass			Reference
Dorsolateral prefrontal cortex (DLPFC)	L:	−42	−34	−20	(Seeley et al., 2007)
	R:	−44	−36	−20	
Lateral posterior parietal cortex (PPC)	L:	−42	−50	−48	(Seeley et al., 2007)
	R:	−46	−54	−42	
Orbitofrontal insula	L:	−40	18	−12	(Seeley et al., 2007)
	R:	−42	10	−12	
Dorsal anterior cingulate cortex (dACC)*	L:	−6	18	30	(Seeley et al., 2007)
	R:	6	22	30	
Superior lateral occipital cortex (sLOC)	L:	−32	−73	38	Harvard Oxford Atlas (Desikan et al., 2006)
	R:	33	−71	39	
Inferior lateral occipital cortex (iLOC)	L:	−45	−76	−2	Harvard Oxford Atlas (Desikan et al., 2006)
	R:	−46	−74	−2	
The ventral striatum (nucleus accumbens)	L:	−9	12	−7	Harvard Oxford Atlas (Desikan et al., 2006)
	R:	9	12	−7	
Dorsal striatum (caudate nucleus and putamen)	L:	−20	4	4	Harvard Oxford Atlas (Desikan et al., 2006)
	R:	21	5	4	
Thalamus	L mediodorsal:	−6	−17	3	Specification of the neurofeedback-related regions by Paret et al. (2018); parcellation by Najdenovska et al. (2018)
	L ventrolaterodorsal:	−16	−21	13	
	R mediodorsal:	5	−15	3	
	R ventrolaterodorsal:	15	−22	−13	
Ventromedial prefrontal cortex (vmPFC)**		−4	56	−6	(Paret et al., 2018)
		2	52	−12	
		−8	58	8	
Left orbitofrontal cortex (OFC)		−18	40	−12	(Paret et al., 2019)

*The two overlapping spheres were joined into a single ROI. **The three overlapping spheres were joined into a single ROI.

When selecting ROIs for the attention hypothesis (see Table 2), we aimed to examine attention-related networks that are consistently identified in the resting-state fMRI sessions and looked to the CONN Networks atlas which was derived by the CONN toolbox developers from the Human Connectome Project data (*N* = 497). From this atlas, we included in the list of ROIs components of the Dorsal Attention Network, those of the Salience Network as related to the selection of salient stimuli and behavioral responses (Menon, 2015), as well as those of the FPCN and the Language Network as engaged in working memory and executive control processing. We also included regions of interest comprising the Visual Network because of the visual nature of the applied neurofeedback. The resulting ROIs are summarized in Table 2.

**Table 2:** ROIs within the attention-related networks (Hypothesis 2).

Network	ROI	MNI coordinates (x, y, z) of the center of mass		
Visual	Primary Visual Cortex	2	−79	12
Visual	Ventral Visual Pathway	0	−93	−4
Visual	Dorsal Visual Pathway L	−37	−79	10
Visual	Dorsal Visual Pathway R	38	−72	13
Salience	Anterior cingulate cortex (ACC)	0	22	35
Salience	Anterior Insula L	−44	13	1
Salience	Anterior Insula R	47	14	0
Salience	Rostral prefrontal cortex (RPFC) L	−32	45	27
Salience	Rostral prefrontal cortex (RPFC) R	32	46	27
Salience	Supramarginal gyrus (SMG) L	−60	−39	31
Salience	Supramarginal gyrus (SMG) R	62	−35	32
Dorsal Attention	Frontal eye field (FEF) L	−27	−9	64
Dorsal Attention	Frontal eye field (FEF) R	30	−6	64
Dorsal Attention	Intraparietal sulcus (IPS) L	−39	−43	52
Dorsal Attention	Intraparietal sulcus (IPS) R	39	−42	−54
Language	Inferior frontal gyrus (IFG) L	−51	26	2
Language	Inferior frontal gyrus (IFG) R	54	28	1
Language	Superior temporal gyrus, the posterior portion (pSTG) L	−57	−47	15
Language	Superior temporal gyrus, the posterior portion (pSTG) R	59	−42	13
Frontroparietal control network	Lateral prefrontal cortex (LPFC) L	−43	33	28
Frontroparietal control network	Lateral prefrontal cortex (LPFC) R	41	38	30
Frontroparietal control network	Posterior parietal cortex (PPC) L	−46	−58	49
Frontroparietal control network	Posterior parietal cortex (PPC) R	52	−52	45

*Note: The masks were derived from the CONN networks atlas*.

#### ROI Analysis: First and Second Levels

During the first-level ROI-analysis, ROI-to-ROI correlation matrices were computed based on the BOLD-signal time series averaged from voxels within each ROI (see Figure 4). Next, at the group level, a GLM model with a two-sided *T*-contrast for post-session vs. pre-session, NF vs. sham-NF (equivalent to an *F*-contrast) was applied to discriminate the effects related to the real but not sham neurofeedback. The number of invalid scans in each participant’s data was included in the model as a covariate of no interest to control for any residual differences in head movements.

A matrix of connectivity differences assessed with the contrasts was further entered into a non-parametric NBS analysis. NBS provides statistical techniques for testing hypotheses about interconnected sets or clusters of connections (networks) rather than individual connections. An analogy may be drawn between the NBS and values derived from clusters on the spatial fMRI activation maps, but with supra-threshold connections in place of activated voxels. For the group comparisons, NBS statistics are computed directly at the level of the group connection matrix without addressing the clusters of connections at the individual subject level. The NBS approach also implements the multiple comparison correction in a way resembling topological (cluster-wise) family-wise error/false discovery rate (FWE/FDR) corrections for the activation maps. In the present study, we looked for any network or set of connections, that showed an aggregated change in connectivity between the pre- and post- sessions that were significantly different between the two groups. The FWE rate of *p* < 0.05 was used for the set of connections (NBS by intensity statistics) given a connection-wise statistical threshold of *p* < 0.01 uncorrected (two-sided).

#### Intrinsic Connectivity Contrast Analysis

For the hypothesis-free ICC analysis, whole-brain voxel-to-voxel correlation matrices were computed for each participant. Based on these matrices, the ICC was computed for each voxel in each participant—a measure that characterizes the overall strength of the functional connectivity between the given voxel and the rest of the brain. The ICC values entered the second-level GLM, and group-level contrasts were obtained. Changes in the connectivity maps after the NF and sham-NF sessions were assessed and compared between groups (NF vs. sham-NF, post- vs. pre- sessions) with an *F*-contrast (two-sided). The number of invalid scans in each participant’s data was included in the model as a covariate of no interest. We looked for the areas of the brain where the difference between the pre- and post-session connectivity was significantly different in the two groups. Multiple comparison control was implemented with a false discovery error rate of *q* < 0.05 at the cluster-level, given a voxel-wise statistical threshold of *p* < 0.005 uncorrected.

## Results

### ROI Analysis: Neurofeedback Contour Hypothesis

**Figure 5:**
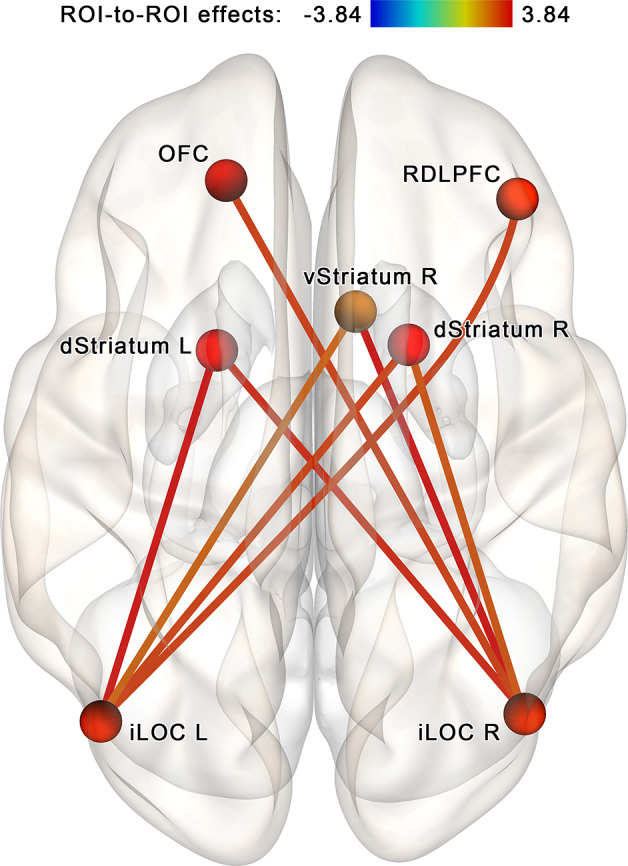
Increased connectivity within the proposed neurofeedback contour after the infra-low frequency NF session. A subnetwork containing theinferior lateral occipital cortex (iLOC), right dorsolateral prefrontal cortex (RDLPFC),left orbitofrontal cortex (OFC), ventral striatum (vStriatum), and dorsal striatum (dStriatum)shows increased connectivity post- vs. pre-, NF vs. sham (*p* < 0.05, FWE-corrected). We propose that these connections reflect the coupling of brain areas targeting the accomplishment of the neurofeedback task.

The NBS analysis performed for the regions of interest within the proposed neurofeedback contour (see Table 1, Figure 4A) identified a subnetwork (a cluster of connections) consisting of 7 nodes and 16 edges (in CONN toolbox implementation, each edge is counted in both possible directions; see Figure 5). The overall intensity of the connections within the subnetwork changed significantly more in the post- vs. pre-session rsfMRI scans in the NF compared to the sham-NF group (*p* < 0.05, FWE-corrected; cluster-defining threshold at *p* < 0.01, uncorrected). The revealed network component reflects the increasing functional connectivity of the bilateral inferior lateral occipital cortex (LOC) with the dorsal and ventral striatum, the right dorsolateral prefrontal cortex (RDLPFC), and the left OFC.

**Figure 6:**
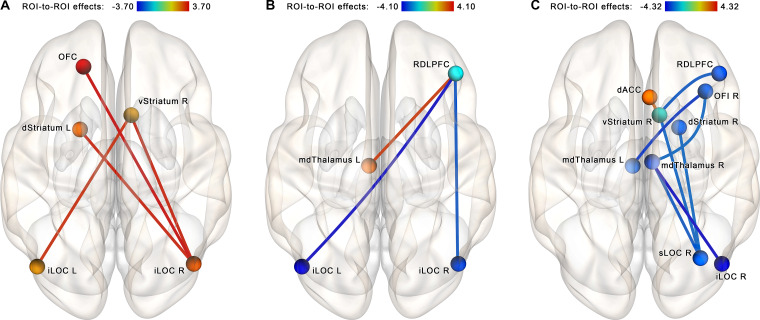
Within-group comparisons, ROI-to-ROI NBS analysis within the proposed neurofeedback contour. **(A)** Post-EEG vs. pre-EEG comparison, NF group. **(B)** Post-EEG vs. pre-EEG comparison, sham-NF group. **(C)** Altered connectivity as a rescanning effect (data from the NYU CSC TestRetest dataset). A strengthening of connections of the lateral occipital cortex with subcortical nuclei and the orbitofrontal cortex is observed in the neurofeedback group, while no such tendency is observed in the Sham and TestRetest groups. The connections are contrasted at the post- vs. pre-, and network-based statistics are used to correct for multiple comparisons (*p* < 0.05, FWE-corrected). Abbreviation key: dACC, dorsal anterior cingulate cortex; iLOC, inferior lateral occipital cortex; sLOC, superior lateral occipital cortex; OFC, orbitofrontal cortex; OFI, orbitofrontal insula; RDLPFC, right dorsolateral prefrontal cortex; dStriatum, dorsal striatum; vStriatum, ventral striatum; mdThalamus, mediodorsal thalamus.

The follow-up NBS analysis performed for each group separately at identical statistical thresholds revealed a set of four increasing connections (right iLOC with left dorsal striatum, right ventral striatum, and left OFC; left iLOC with right ventral striatum) in the NF group (see Figure 6A). In the control group, another set of three connections that became altered after a sham-NF session was identified. This subnetwork included decreasing connectivity between the RDLPFC and the iLOC bilaterally and increasing connectivity between the RDLPFC and the left mediodorsal thalamus (see Figure 6B).

To make inferences about the possible effects of the sham-NF in the control group of our study, we reproduced the same analysis for the NYU CSC TestRetest dataset. In the TestRetest group, comparing the second vs. first scanning, we revealed a cluster of regions with decreased connectivity, including the LOC, RDLPFC, right orbitofrontal insula, and subcortical nuclei, along with increased connectivity of the anterior cingulate cortex (ACC) with the nucleus accumbens (see Figure 6C). Notably, the opposite tendency was observed after the neurofeedback in our study.

### ROI Analysis: Attention Hypothesis

**Figure 7:**
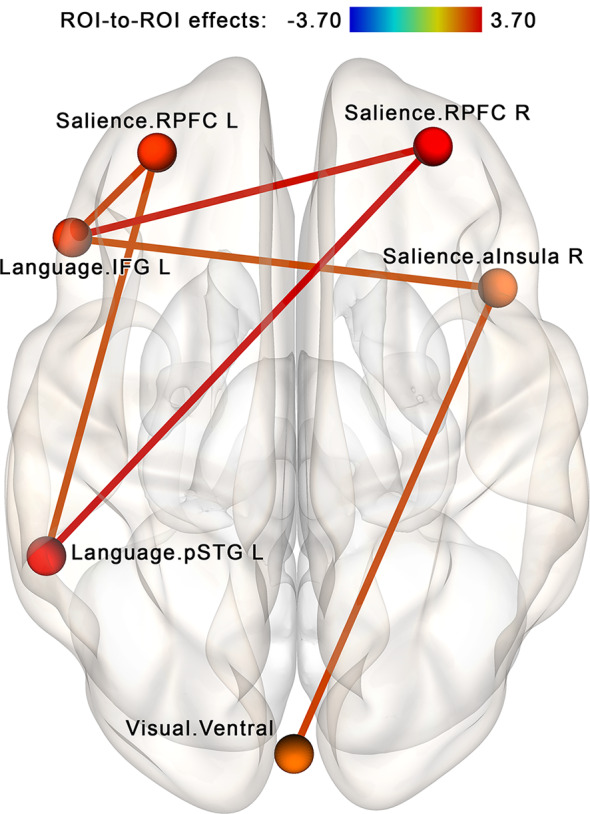
Increased connectivity between the salience, language and visual networks after the infra-low frequency NF session. A subnetwork containing the right and left rostral prefrontal cortex (RPFC), left inferior frontal gyrus (IFG L; i.e., Broca’s area), left posterior portion of the superior temporal gyrus (pSTG L; i.e., Wernicke’s area), right anterior insula (aInsula R) and ventral visual pathway shows increased connectivity post- vs. pre-, NF vs. sham (*p* < 0.05, FWE-corrected). We propose that these connections reflect the desired effects of the neurofeedback: an integrative tendency toward multimodal processing.

The NBS analysis performed for the regions of interest within the attention-related networks (see Table 2, Figure 4B) identified a subnetwork consisting of 6 nodes and 12 edges (see Figure 7). The overall intensity of the connections within this network component changed significantly more in the post- vs. pre-rsfMRI sessions in the NF compared to the sham-NF group (*p* < 0.05, FWE-corrected; individual connections thresholded at *p* < 0.01, uncorrected). The revealed subnetwork reflects the increasing functional coupling of the key salience network areas with the ventral visual stream and the left-sided language areas.

**Figure 8:**
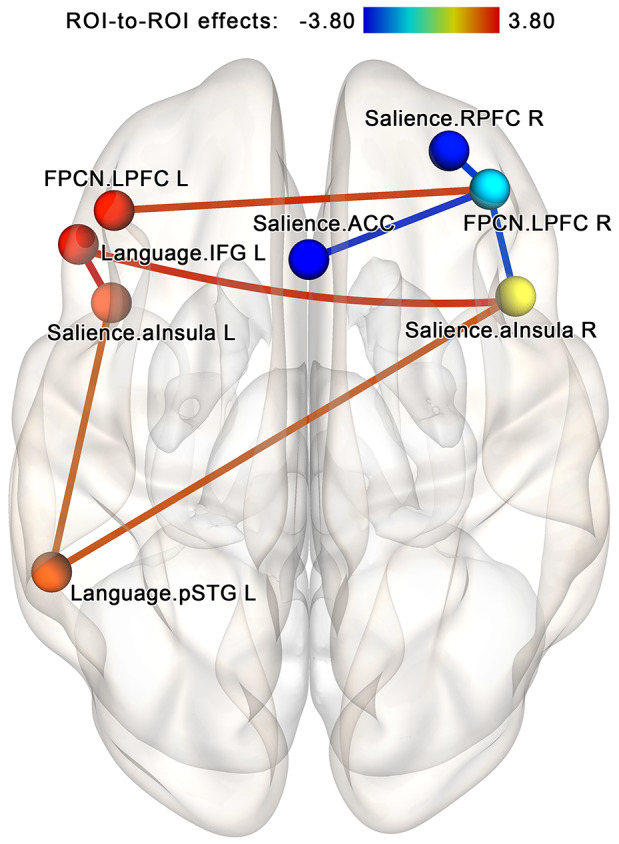
Within-group comparison for the neurofeedback group; analysis within the components of the attention-related networks. In the neurofeedback group, increased connectivity within the regions of the salience and language networks is observed after the session. This effect is similar to the core findings of the NF vs. sham analysis. The connections are contrasted at the post- vs. pre-, and network-based statistics are used to correct for multiple comparisons (*p* < 0.05, FWE-corrected). Abbreviation key: ACC, anterior cingulate cortex; aInsula, anterior insula; FPCN, frontoparietal control network; IFG, inferior frontal gyrus; LPFC, lateral prefrontal cortex; pSTG, superior temporal gyrus, posterior portion; RPFC, the rostral prefrontal cortex.

The follow-up analysis performed for each group separately showed that in the NF group, the post-NF vs. pre-NF contrast at the same NBS subnetwork threshold by intensity identifies a cluster of 16 edges (eight bidirectional connections) involving increasing connectivity within a group of two nodes from the salience network (left and right anterior insula) and two nodes from the language network (left superior temporal gyrus and inferior frontal gyrus ROIs)—see Figure 8. Additionally, the revealed subnetwork included a strengthening connection between the lateral prefrontal cortex regions in both hemispheres (a part of the frontoparietal network) as well as degrading connectivity between the right lateral prefrontal cortex and three regions from the salience network (anterior insula bilaterally and the right rostral prefrontal cortex). No subnetworks significantly altered by the sham-NF session were found in the control group. However, at low statistical thresholds (*p* < 0.05 uncorrected for multiple comparisons), there was a noticeable tendency for decreasing connectivity between the components of the FPCN on the one hand and components of the salience and language networks on the other hand.

In the TestRetest sample of the NYU CSC, repeated scanning did not alter the connectivity within attention-related networks.

To further explore our findings in terms of network interactions, we compared possible intrinsic connectivity modifications both within each of the three affected networks (salience, language, and visual) and between the salience network and each of the two other networks. For this purpose, average connectivity values for all ROI pairs within the same network and all between-network ROI pairs (i.e., each ROI with every ROI in the other network, but not its network) were computed for each participant and used for a second-level analysis. We found no significant within-network connectivity changes in post- vs. pre-session scans in the salience, language, or visual networks. However, between-network connectivity significantly increased in the NF vs. sham-NF group, at the post- vs. pre-session for both tested network pairs (two-sided *t* = 2.65, *p* = 0.01 for salience and language networks; *t* = 2.86, *p* = 0.006 for salience and visual networks). These significant between-network connectivity modifications were observed in the NF group (two-sided *t* = 2.16, *p* = 0.04 for salience and language networks; *t* = 3.26, *p* = 0.03 for salience and visual networks), but not the sham-NF group (two-sided *t* = −0.73, *p* = 0.79 for salience and language networks; *t* = −0.24, *p* = 0.59 for salience and visual networks). Thus, the network coupling is related to the neurofeedback itself rather than to any placebo effect.

### *Post hoc* ROI Analysis: Interaction of the Two Revealed Subnetworks

When analyzing the study results, it is reasonable to ask whether the two sets of findings (see Figures 5, 7) are interrelated. To address this question, we performed a between-network ROI connectivity analysis for the two sets of ROIs revealed by the NBS approach for the neurofeedback contour (seven ROIs, see Figure 5) and attention hypotheses (six ROIs, see Figure 7). Between-network connectivity significantly increased in the NF vs. sham-NF group, at post- vs. pre-session (*t* = 4.55, *p* < 0.001 two-sided). Notably, such connectivity modifications were observed only in the NF group (*t* = 4.09, *p* < 0.001 two-sided), not in the sham-NF group (t = −0.97, *p* = 0.262 two-sided). Thus, the two branches of results are indeed connected, and luckily represent the same phenomena—the implicit infra-low frequency EEG neurofeedback process.

### Intrinsic Connectivity Contrast

The whole-brain voxel-to-voxel exploratory analysis revealed no changes in the ICC values associated with the NF vs. sham-NF sessions. However, this null finding might be due to insufficient statistical power or to an inappropriate choice of the whole-brain connectivity metric.

## Discussion

When designing the study, we hypothesized that the transient neuronal assembly formed to deal with neurofeedback may be detectable immediately after the session using the rsfMRI. Our findings from the network-based analysis met this expectation. We identified the network related to implicit infra-low frequency EEG neurofeedback, consisting of the right RDLPFC, left OFC, bilateral LOC, right ventral striatum, and bilateral dorsal striatum (see Figure 5). The involvement of the lateral prefrontal and the secondary visual cortices reflects control over the neurofeedback process. The lateral prefrontal cortex is well known to be the key area in top-down control. In particular, it enhances the selectivity of representations in the sensory cortex throughout working memory maintenance (Sreenivasan et al., 2014). Taking into account the modality of the feedback that was used (visualization of a moving rocket), we believe that the role of the secondary visual cortex was the extraction of the feedback information—the speed of the rocket. This explanation is in line with current neuroscientific models of neurofeedback (Gaume et al., 2016). Involvement of the right but not the left DLPFC is a less expected finding, and we think it may be related to the implicit paradigm of the training. According to other studies, the right hemisphere is thought to be more involved in implicit vs. explicit learning (Davidson and Hugdahl, 1995). Further studies of implicit neurofeedback are necessary to prove or invalidate this hypothesis.

Areas of the salience network with a known role in explicit neurofeedback, the anterior insula, and ACC, were not incorporated into the revealed network, in contrast with the striatum. Our findings are in agreement with data from fMRI neurofeedback studies: while the salience network is responsible for the perception of conscious reward (Emmert et al., 2016), the unconscious reward is thought to be mediated by the striatum (Ramot et al., 2016). Moreover, according to the recent study by Paret et al. (2018), even in explicit neurofeedback the ventral striatum rather than the insula may process the reward. The involvement of the left OFC is also in line with observations of Paret et al. (2019), who described its role in the monitoring of feedback congruence on the model of explicit fMRI neurofeedback. Thus, a special neural contour including the right RDLPFC, left OFC, bilateral LOC, right ventral striatum, and bilateral dorsal striatum is formed during an implicit EEG neurofeedback session.

Another group of results includes increased connectivity between the salience, language, and visual networks (see Figure 7). The salience network performs the integration of multimodal information (anterior insula) and selection of salient stimuli with contributions to high-order abstract thinking (rostral prefrontal cortex; Dumontheil, 2014; Menon, 2015). The anterior insula is especially important in interoception (Schulz, 2016). Increased connectivity between the networks responsible for processing different types of information—interoceptive, verbal, and visual—may reflect high-order integration in multisensory processing. This finding is in line with observations of alert states with increased awareness of external and internal stimuli resulting from infra-low frequency neurofeedback sessions at the T4P4 placement (Othmer, 2017).

Since we found some effects in the sham-NF condition as well as in the NF condition, we used the data from the TestRetest group from the NYU CSC dataset to test for possible effects of the MRI scanning repetition and to disentangle these effects from the sham-NF effects *per se*. Decreased connectivity was found among the hypothetical NF circuit regions in the retesting condition (NYU CSC dataset) and to some extent in the sham-NF condition (our study’s data). This finding supports the idea that adaptation to the MRI settings along with the monotony of repeated scanning results in a shift towards a more passive state. Notably, the NF group demonstrated increased connectivity that may be treated as functional integration.

The observed neurophysiological effect—functional integration of the large-scale brain networks involved in sensory processing—is of potential practical importance. The ability to integrate multisensory signals is associated with higher emotional intelligence (Perepelkina et al., 2017) and lower levels of alexithymia (Grynberg and Pollatos, 2015) and thus is essential for successful social functioning. The perception of emotions relies on different modalities: we see facial expressions, hear words and intonations, feel our physiological state (calm or increased arousal), get proprioceptive impulses about posture—all these sources contribute towards the decoding of emotional states (Critchley and Garfinkel, 2017; Reschke et al., 2018). Taken together, we think that the observed increase in connectivity between the salience, language, and visual networks after infra-low frequency neurofeedback from the T4P4 site may represent a beneficial effect of potential clinical value.

The current study has limitations and raises several questions for further research. First, the ROI analysis, including the division into the neurofeedback contour and attention hypothesis, was based on the *a priori* assumptions coming from the literature. Relying on the previously developed neuroscientific models of neurofeedback (Gaume et al., 2016; Sitaram et al., 2017), we attempted to evaluate how the implicit EEG neurofeedback may fit into the existing theoretical framework. Second, since we assessed the immediate effects of a single session, the stability of the detected changes in brain connectivity is uncertain. Third, there are limitations of the sham-based control (Lubianiker et al., 2019): since the control group did not receive another type of learning, we are unable to make inferences regarding the specificity of the revealed connectivity changes for the particular neurofeedback task, including the role of the implicit vs. explicit design. However, the study findings have clear similarities with the observations coming from implicit fMRI neurofeedback (Ramot et al., 2016). Fourth, the fMRI connectivity was the only outcome measure, since EEG measures that are sensible for the effects of implicit infra-low frequency neurofeedback are not yet described. This neurofeedback modality fails to fit into the operant conditioning and, in general, into the behavioral model: there is no “target” and no “training.” The development of the theoretical framework for implicit neurofeedback requires additional research.

Further research may address the remaining questions. To test the stability of the revealed neurofeedback effects, studies including functional neuroimaging before and after a full course of neurofeedback are feasible. It is also necessary to include in further studies relevant measures of clinical efficacy, which would allow for correlation of the observed effects with the changes in brain connectivity. Next, it is reasonable to compare different neurofeedback modalities, including implicit vs. explicit, in terms of neuronal mechanisms, since the existing research does not allow us to separate general and modality-specific alterations in brain connectivity.

Despite the existing uncertainties, the study adds an optimistic note to the discussions around neurofeedback. We have shown for the first time that even a single implicit EEG neurofeedback session can result in significant modulation of the brain’s intrinsic connectivity. Importantly, the randomized sham-controlled design with standardized procedures and instructions allowed us to discriminate against the influence of intervention *per se* from placebo effects. Our study contributes to the neuroscientific understanding of the mechanisms of neurofeedback and shows several directions for further research.

## Data Availability Statement

The dataset for this study can be found in OpenNeuro repository, reference number ds001408. DOI: http://doi.org/10.18112/openneuro.ds001408.v1.0.2. URL: https://openneuro.org/datasets/ds001408/versions/1.0.2.

## Ethics Statement

The studies involving human participants were reviewed and approved by Inter-University Ethics Board of Moscow. The participants provided their written informed consent to participate in this study.

## Author Contributions

OD performed the neurofeedback. RV, AR, LL, and EP collected the fMRI data. EP, RV, OD, EM, and VS performed the data analysis. All authors contributed to the design of the study and the writing of the manuscript.

## Conflict of Interest

The authors declare that the research was conducted in the absence of any commercial or financial relationships that could be construed as a potential conflict of interest.

## Footnotes

^1^http://www.fil.ion.ucl.ac.uk/spm

^2^http://www.nitrc.org/projects/conn

^3^http://www.nitrc.org/projects/artifact_detect
